# Speaker differences in volitional voice modulation reflected in empathy and functional activation patterns

**DOI:** 10.1371/journal.pone.0325207

**Published:** 2025-07-28

**Authors:** Stella Guldner, Frauke Nees, Herta Flor, Carolyn McGettigan

**Affiliations:** 1 Department of Child and Adolescent Psychiatry and Psychotherapy, Central Institute of Mental Health, Medical Faculty Mannheim, Heidelberg University, Germany; 2 Institute of Cognitive and Clinical Neuroscience, Central Institute of Mental Health, Medical Faculty Mannheim, Heidelberg University, Mannheim, Germany; 3 Institute of Medical Psychology and Medical Sociology, University Medical Center Schleswig- Holstein, Kiel University, Kiel, Germany; 4 Department of Speech, Hearing and Phonetic Sciences, University College London, London, United Kingdom; Sapporo Gakuin University, JAPAN

## Abstract

How we use our voice is central to how we express information about ourselves to others. A speaker’s dispositional social reactivity might contribute to how well they can volitionally modulate their voice to manage listener impressions. Here, we investigated individual differences in social vocal control performance in relation to social reactivity indices and underlying neural mechanisms. Twenty-four right-handed speakers of British English (twenty females) modulated their voice to communicate social traits (sounding likeable, hostile, intelligent) while undergoing a rapid-sparse fMRI protocol. Performance in social vocal control was operationalized as the specificity with which speakers evoked trait percepts in an independent group of naïve listeners. Speakers’ empathy levels, as well as psychopathic and Machiavellian traits, were assessed using self-report questionnaires. The ability to express specific social traits in voices was associated with activation in brain regions involved in vocal motor and social processing (left posterior TPJ, bilateral SMG, premotor cortex). While dispositional cognitive empathy predicted general vocal performance, self-reported levels of Machiavellianism were specifically related to better performance in expressing likeability. These findings highlight the psychological and neural mechanisms involved in strategic social voice modulation, suggesting differential processing in a combined network of vocal control and social processing streams.

## Introduction

The human voice is a predominantly social signal and the primary channel for social communication. Listeners form impressions about a speaker rapidly [1] and reliably [2], and these impressions guide subsequent behavior towards the speaker [3–7]. Speakers can influence listener impressions about themselves by modulating their voice, for instance to sound more dominant, intelligent or attractive [8,9]. Using the voice effectively to achieve favourable trait judgments from listeners might be an important tool in social interactions. Nevertheless, speakers differ considerably in their ability to modulate their voice on demand [10]. So, what are the speaker characteristics that influence volitional voice modulation?

One important route might be a speaker’s ability to empathize with an interlocutor, i.e., to understand their feelings and thoughts, in order to adjust their own behavior accordingly. On the listener’s side, higher levels of empathy seem to support the *decoding* of nonverbal social information in vocalizations, such as authenticity [11], irony [12,13], or emotions [14]. However, speakers also spontaneously modulate their voice to *encode* information fitting to the listener’s needs [15,16]. As such, empathy might also support the speaker in strategic voice modulation, helping them to modulate the voice in accordance with the social context. Empathy includes both cognitive and affective aspects. Cognitive empathy in particular might be central to volitional voice changes. It is thought to be under voluntary control [17] and closely related to theory of mind (ToM) [18]. In populations with specific impairments in cognitive empathy, vocal behaviour is often rigid or emotionally flat [19–21]. On the other hand, people with affective social reactivity deficits but often preserved cognitive empathy capacity, such as Machiavellianism or Psychopathy [22], show both a strategic use of linguistic vocal behaviour in social interactions [23] and proficieny in volitional facial expression of affect [24]. This might be specific to volitional behaviour, as other work shows that psychopathic traits are associated with less spontaneous prosodic modulation of affective words [21]. Machiavellianism is characterized by a strategic, self-serving manipulative interpersonal style, whereas psychopathy is associated with a highly unemotional, callous, and impulsive style [25,26]. Thus, the ability to coolly apprehend others’ feelings and thoughts might be a route through which social opportunists manage others’ impressions.

On the neural level, voluntary voice changes are achieved by a network of regions involved in vocomotor control – the vocomotor network (VMN) – that includes left inferior frontal gyrus (IFG), anterior cingulate cortex (ACC), supplementary motor cortex (SMA), supramarginal gyrus (SMG), superior termporal gyrus (STG), insula, basal ganglia (BG) and cerebellum (reviewed by [27,28]. Together with sensorimotor cortices, activity patterns in these areas are associated with task performance in the imitation or production of novel speech sounds [29–32], pitch control in singers (particularly sensorimotor cortices [33]), and voice training in singers [34]. Very few studies have investigated inter-individual differences in *social* voice modulation on the neurophysiological level. One study found the ability to volitionally modulate the voice to express sad or happy emotions is associated with increased functional activation of right IFG (triangular part), right middle frontal gyrus, and left superior frontal gyrus that might be related to motor planning for prosody production [14]. Together, these studies suggest that differences in functional activation in VMN might underlie volitional voice modulation efficacy. Beyond the VMN, precuneus, medial prefrontal cortex (mPFC), and superior temporal sulcus (STS)/Temporo-parietal junction (TPJ) support the volitional expression of explicit social traits in the voice [9], as well as explicit [35] and covert [36] vocal identity expression. These areas are often referred to as the social brain network, since they are reliably activated together during domain-general social cognitive processing tasks [37]. Interestingly, activation in STS/TPJ and bilateral insulae during prosody *perception* was negatively associated with psychopathy traits, while activation in left IFG was associated with listeners’ affective empathy levels [14]. However, whether differences in activation in these regions also applies to vocal *production* remains to be tested.

Importantly, most previous studies have used either combined measures of empathy [12,13], focussed on affective empathy components [11,14] or studied individual differences in empathy in relation to perception but not voice production. Moreover, these studies mostly refer to emotional, but not social voice changes. It is therefore unclear how individual differences in social reactivity might contribute to the ability to volitionally express social information through the voice, and which neurophysiological mechanisms might support this ability. Here, we addressed this gap and hypothesized, first, that performance in social vocal control would be positively associated with empathy (particularly cognitive empathy) and socially-opportunistic traits (Machiavellianism and psychopathic traits). Given that these measures are interrelated, we tested this in the framework of multiple regression models, to determine each individual and unique contribution. Second, we probed the underlying neurophysiological networks associated with individual differences in social vocal control, hypothesizing a positive association between task performance and activation in areas associated with domain-general social processing (mPFC, STS/TPJ, precuneus). Finally, we investigated whether speaker traits – regardless of their relationship to behavioural performance – predicted underlying differences in task-relevant functional activations during social voice modulation.

## Methods

Here we present novel individual-differences analyses using functional MRI and speech production data previously described with group-level analyses [9]. Data relating to individual social reactivity and trait scales are being reported and analysed here for the first time.

### Speakers

Twenty-four right-handed, native British English speakers (M_age_ = 21.04 SD = 3.26, 3 male) participated in this experiment. Data collection occurred from 31.10.2017 to 13.12.2017. All speakers had normal or corrected-to-normal vision and reported no history of hearing, language, neurological, or psychiatric disorders and were recruited from the participant pool at the Department of Psychology at Royal Holloway, University of London, and received 30 GBP as reimbursement. All speakers provided their full informed and written consent prior to participation according to the Declaration of Helsinki (1991). This study was approved by the research ethics committee of Royal Holloway, University of London (587-2017-10-24-14-50-UXJT010).

### Assessment of empathy, machiavellianism and psychopathy

To measure individual differences in trait empathy, speakers completed the Questionnaire of Cognitive and Affective Empathy (QCAE) [38]. The QCAE is a 31-item questionnaire measuring cognitive empathy and affective empathy aspects. Speakers choose their level of agreement with each item from 1 (*strongly disagree)* to 4 (*strongly agree*). The sum score ranges from 19 to 76 for cognitive empathy and 12–48 for affective empathy. To assess traits of psychopathy and Machiavelliansim, we used the Short Dark Triad (SD3) [39], a 27-item self-report questionnaire. We focused on dispositional psychopathy and Machiavellianism, as these constructs are highly associated with social manipulative behaviours. Responses are given in agreement with each item on a 5-point Likert-scale with anchors 1 (*strongly disagree*) to 5 (*strongly agree*). Sumscores for each scale range from 9 to 45.

### Social vocal control task

The main experimental task consisted of a social vocal control task in which speakers were asked to express social and non-social traits in the voice. Social traits included vocally expressed intelligence, likeability, and hostility. Modulating the voice to convey a large body size, as well as speaking in non-modulated neutral voice, were implemented as control conditions (for detailed instructions on trait expressions see [9]). Exemplars consisted of four two-syllable, five-letter pseudowords with a C-V-C-V-C (C = consonant, V = vowel) phonotactic structure (*belam*, *lagod*, *minad*, and *namil*; [40]).

### Design and procedure

Speakers filled out the self-report questionnaires online prior to the fMRI scanning session. On the scanning day, speakers first received a brief training on the vocal control task before completing the task in the MRI scanner over the course of 4 runs. Each run consisted of a randomized order of the 5 vocal modulation conditions (neutral/ large/ hostile/ likeable/ intelligent) paired with one of the 4 exemplars, of which each combination appeared during 3 Go trials and 3 No-Go trials. Go and No-Go trials were presented in randomized order. In total, each run included 150 trials, of which 30 were rest trials.

Both Go and No-Go trials started with a two-second presentation of the target trait and a fixation cross. During Go trials, the fixation cross was then substituted with an exemplar for 1.5 seconds. During this 1.5 sec silent gap speakers were asked to vocalize the exemplar while expressing the target trait. Recordings were made on an in-scanner MR-compatible microphone (Opto-acoustics, FOMRI-III). During No-Go trials, the fixation cross remained on the screen for the duration of the silent gap and no exemplar was presented. Go and No-Go trials, therefore allowed filtering out neural activation specifically related to ongoing voice production. Visual cues were projected onto a screen at the back of the scanner bore and viewed via a mirror on the head coil. The total scanning time was approximately 50 minutes.

Speakers were re-invited approximately 1 week later for performance ratings on their in-scanner recordings. They were asked to rate the trait intensity on a 7-point Likert scale ranging from 1 (not at all) to 7 (very much) for each of their voice recordings blocked by the expressed target trait in a soundproof booth via Sennheiser Headphones HD306. We selected one recording for each modulation condition and for each speaker that had received the speaker’s maximal rating. This was done to ensure that independent evaluations of performance would be based on sounds where the speakers had felt confident in their expression of the target trait.

### Naïve ratings

**Fig 1:**
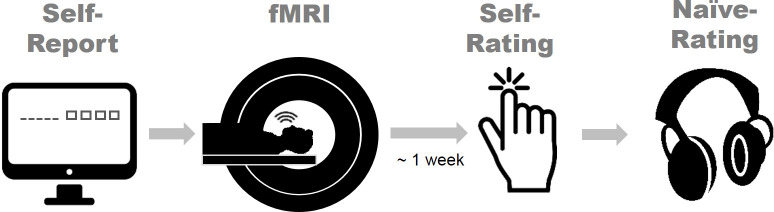
Experimental procedure.

For each speaker, the highest-rated recording for each trait category was intensity normalized across speakers [9] and then presented to 24 naïve listeners. All raters (M_age_ = 19.92, SD = 1.47, 4 male) were recruited at the Department of Psychology at Royal Holloway, University of London, gave their informed consent before participation and received monetary compensation for their participation of 5 GBP. To reduce the experimental duration, each listener heard the recordings of a subset of 10 speakers while ensuring that each speaker was heard by at least 10 different listeners. For each speaker, we included one recording of each vocal modulation condition (trait) and one recording of their neutral voice. Each listener heard and rated each recording of each speaker on all trait scales in separate blocks. Each block consisted of one social trait rating for all recordings in randomized order (for details see [9]). Listeners indicated their responses on 7-point Likert scales, with anchors at 1 (*not at all*) to 7 (*very*) to rate how strongly a voice expressed a given trait. Block order was randomized across raters. Raters heard the recordings over Sennheiser Headphones (Sennheiser U.K. Ltd, Marlow, UK) in a soundproof booth (see Fig 1). All in scanner-stimuli, as well as stimuli for rating experiments were presented using the Psychophysics Toolbox [41] in Matlab (2014a, the Mathwork, Natick, MA).

#### Individual differences in social vocal control.

The ratings data were analysed in R (http://www.R-project.org/). To assess the success of the voice manipulations to express social traits, we calculated the average change in naïve ratings for modulated voices relative to the neutral voice samples, for each speaker and each intended trait (i.e., comparing “intelligent” ratings for the neutral and “intelligent” trials), thus allowing to compute a performance index for each trait for each speaker (henceforth ∆ - [trait]).

**Fig 2:**
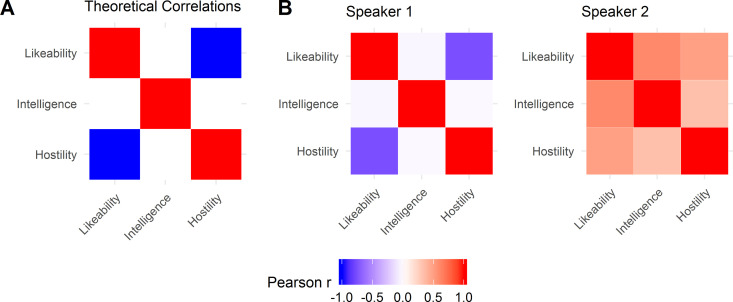
A. Theoretical Representational Similarity Matrix (RSM), showing expected correlations between evoked trait ratings. B. Example RSMs for two speakers, who showed high (left) and low (right) specificity between evoked trait ratings.

Next, we computed representational similarity matrices (RSM) based on the pairwise Pearson correlation coefficients of naïve ratings between pairs of the three social traits (likeability, hostility and intelligence) for each speaker. These matrices permit us to characterise the similarity in perceptual mental representations between different stimulus categories [42]; see, e.g., [43,44]. In this study, we made use of this analysis to explore how specific voice modulations were expressed in respect to the trait percept they induced (reflected in the naïve ratings). A cell of each speaker’s RSM contained the pairwise correlations of ten listener ratings for the respective two traits, that is, ten ratings (one from each listener) for each of the three trait recordings. From these matrices, we computed a general performance parameter to capture the specificity of social voice modulations for each speaker, i.e., the social voice modulation index. This parameter was estimated by calculating the Euclidean distance (ED) of each speaker’s RSM to a theoretical matrix with maximized discrimination between trait ratings (see Figs 2 and S1). Based on the social space dimensions shown elsewhere [1,9], we assumed that there would be no significant correlations (r = 0) of intelligence ratings with either likeable or hostile voice modulation, and that the ratings for hostile and likeable voices would be anti-correlated (r = −1).

#### Effects of social reactivity on performance in social vocal control.

To investigate the effect of social reactivity indices (cognitive and affective empathy, psychopathic and Machiavellian traits) on vocal modulation performance, we calculated a multiple regression model to test whether social reactivity indices are predictive of social vocal control ability (social voice modulation index). The model included the scaled social reactivity indices as regressors, and age and sex as covariates of no interest. Two participants showed significant influence on the prediction efficacy of the linear model, reflected in multivariate outlier tests on the studentized residuals (uncorrected *p*s < .05), which did however, not reach significance at the Bonferroni-corrected level (*p*s > .08). However, the tests for a bias-inducing residual error distribution was significant (*p* < .001), thus we computed OLS linear regression while excluding these two data points (please note diverging results on the whole sample using OLS here: [45]). For completeness, we also conducted robust regression analysis on the whole sample (i.e., including these influential data points) using an M-estimator. For the robust regression analysis, we used the *robust* package in R; https://CRAN.R-project.org/package=robust). On the trait level, we calculated partial Pearson correlation coefficients pairwise for each spoken trait performance (∆ - [trait]; e.g., ∆-intelligence) and each social reactivity index, controlling for age and sex. We corrected for multiple comparisons using the False Discovery Rate (FDR; [46]).

### Neural correlates of performance in social vocal control

Functional brain images were preprocessed and analysed as described previously in detail [9]; see S1 Text). To determine brain regions associated with performance in social vocal control, we ran a random effects multiple regression model on the group level for the contrast “Social Go> Rest” with the social voice modulation index (ED) as a covariate of interest.

#### Effect of social reactivity on task-based functional activation.

To explore whether differences in behavioural performance related to social reactivity were associated with differences in neural processing during social voice modulation, we computed a whole-brain multiple regression model with social reactivity indices as predictors on functional activation patterns during social voice modulation (Social Go > Rest). In addition, we calculated a multiple regression model based on the behavioral association on the contrast “[trait] Go> Rest” and the social reactivity indices as covariates of interest. All models included a constant intercept, and age and sex as covariates of no interest.

For all imaging analysis, we used a significance threshold of *p* < .001 (uncorrected) at the voxel level and corrected for multiple comparisons at the cluster level with *p* < .05 using an individual cluster extent threshold determined for each contrast using a Monte-Carlo simulation with 1000 iterations [47]. Resulting clusters were labelled based on the location of each peak activation using the built-in Neuromorphometrics and the automated anatomical labeling (AAL) atlas in SPM12.

## Results

### Individual differences in social vocal control

Descriptive statistics of the behavioural measures can be found in Table 1. Performance in social vocal control (i.e., social voice modulation index as ED from theoretical RSM) ranged from 0.25 to 1.64 (M = 0.74, SD = .034), where smaller values denote higher specificity in vocally evoked trait ratings in naïve listeners (see S1 Fig). Cognitive empathy scores ranged from 39 to 76 (M = 57.42, SD = 8.32), affective empathy scores ranged from 22 to 44 (M = 36.67, SD = 5.15), psychopathy trait scores ranged from 10 to 25 (M = 16.92, SD = 3.86) and Machiavellian trait scores varied between 17 and 34 (M = 25.29, SD = 4.79).

**Table 1:** Descriptive statistics of social reactivity and performance in social vocal control and partial Pearson correlation coefficients, controlling for speaker sex and age.

Instrument (range limits)	M (SD)	α	1	2	3	4	5	6
1 Machiavellianism (9–45)	25.29 (4.79)	0.74	–					
2 Psychopathy (9-45)	16.92 (3.86)	0.61	**0.60** ^*^	–				
3 Cognitive Empathy (19-76)	57.42 (8.32)	0.91	0.06	−0.30	–			
4 Affective Empathy (12–48)	36.67 (5.15)	0.81	−0.27	−0.19	−0.00	–		
5 ∆-Hostility	2.07 (1.41)	–	0.08	0.10	−0.01	−0.07	–	
6 ∆-Intelligence	0.53 (0.87)	–	0.13	0.33	−0.32	−0.03	−0.10	–
7 ∆-Likeability	1.30 (0.66)	–	**0.66** ^**^	0.53^t^	0.20	0.01	−0.19	0.05
Social Voice Modulation Index (ED)	0.74 (0.03)	–						

* *p* ≤ .05

** *p* ≤ .01 FDR corrected, ^t^ *p* = .08 FDR corrected; FDR = False discovery rate, M = Mean, SD = Standard deviation; α = Cronbach’s alpha, ED = Euclidean Distance.

On the trait level, speakers evoked a mean increase of 1.30 points on the 7-point Likert-scale with their likeable voice modulations relative to their neutral voice (∆-Likability: M = 1.30, SD = 0.66, *t*(23)=5.64, *p* < .001), an increase of 2.07 for hostile voice (∆-Hostility: M = 2.07, SD = 1.41, *t*(23)=6.68, *p* < .001), and an increase of 0.53 for in*t*elligent voice modulations (∆-Intelligence: M = 0.53, SD = 0.87, *t*(23)=1.87, *p* < .05; results of mul*t*ivariate trait rating comparisons have been reported previously; [9]).

### Effects of social reactivity on performance in social vocal control

The OLS multiple regression model showed that cognitive empathy significantly influenced performance (*β* = −.45, *t* = −3.13, *p* < .01), above all other social reactivity indices (*R*^2^_adj_ = 0.38, *F*(6,15)=3.17, *p* < .03, see Table 2; Results from robust regression, using M-estimator: cognitive empathy (*β* = −.59, *t* = −4.12, *p* < .001), psychopathic *t*raits (*β* = −.70, *t* = −2.92, *p* < .01), and Machiavellian traits (*β* = .56, *t* = 2.47, *p* < .05) predicted the social voice modulation index (ED), mul*t*iple-*R*^2^ = 0.26, RSE = 0.47).

**Table 2:** Results from OLS Regression Model of Social reactivity indices prediction social voice modulation index (ED).

Predictor	*β*	SE	*t*	*p*	Adj. *R*²	*F* (df)
Intercept	0.50	1.00	0.50	0.62		
Machiavellianism (SD3)	0.31	0.20	1.60	0.13		
Psychopathy (SD3)	−0.31	0.19	−1.64	0.12		
Affective empathy (QCAE)	−0.15	0.13	−1.13	0.28		
Cognitive empathy (QCAE)	−0.45	0.14	−3.13	0.01^**^		
Sex (male)	0.50	0.42	1.19	0.25		
Age	−0.04	0.05	−0.80	0.43		
					0.38	3.17^*^ (6,15)

* *p*≤.05

** *p*≤.01; SE = standard error, ED = Euclidean Distance.

We next explored associations between social reactivity and performance in social vocal control on the trait level and found a significant positive association between ∆-Likeability and Machiavellianism (*r*_*p*_ = .66, *p* < .02), but no other traits (all *r*s < .32, all *p*s > .08, see Table 1). To explore this result further, we employed an additional multiple regression model including ∆- Likeability as the outcome variable and all social reactivity indices as predictor variables, while controlling for age and sex. The model explained 46% of the variance in ∆-Likeability (*R*^2^_adj_ = .46, *F*(6,17)=4.21, *p* < .01) and revealed that levels of Machiavellianism predicted likeable voice performance independent of other social reactivity indices (*β* = .54, *t*(17)=2.30, *p* < .05).

### Neural correlates of performance in social vocal control

**Fig 3:**
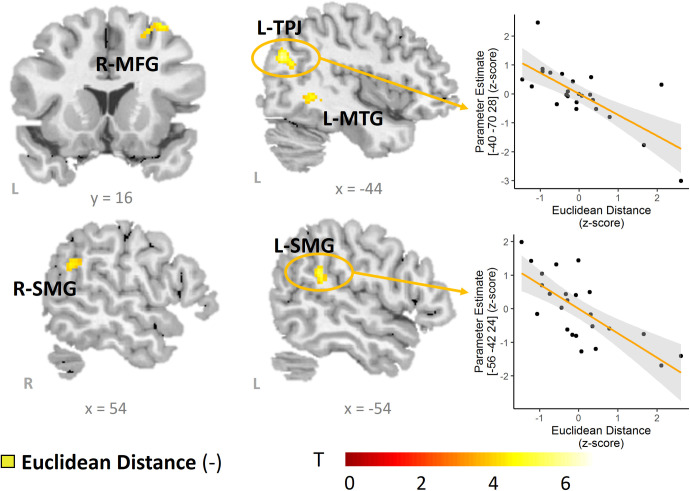
Correlates of social vocal control ability: activation maps. The multiple regression model revealed a significant negative association between the social voice modulation index (ED) and functional activation in response to Social Go trials > Rest in left posterior TPJ, MTG and right MFG, as well as bilateral SMG. Associations are illustrated based on the peak-voxel parameter estimates in left posterior TPJ and SMG. MFG = middle frontal gyrus, MTG = middle temporal gyrus, TPJ = temporo-parietal junction, L = left, R = right.

The whole-brain multiple regression model showed a significant association between functional activation and social voice modulation index (ED) in 9 clusters (uncorrected *p* < .001, *k* = 61) with peak voxels in left posterior temporo-parietal junction (TPJ), right middle frontal gyrus (MFG), bilateral supramarginal gyrus (SMG), left middle temporal gyrus (MTG), left somatosensory cortex in postcentral gyrus (S1), cuneus, and bilateral caudate (see Table 3 and Fig 3).

**Table 3:** Functional activations for the multiple regression whole-brain model of performance (social voice modulation index, ED) on Social Go > Rest. All contrasts are negative correlations (i.e., greater activation with lower social voice modulation index); the positive direction showed no significant voxels.

Contrast	*k*	Region	Hem.	x	y	z	*T*	*Z*
(-) social voice modulation index on Social Go > rest	338	Caudate	L	−18	−22	22	6.31	4.63
			L	−8	−26	16	4.77	3.85
	295	Posterior Temporo-Parietal Junction	L	−40	−70	28	5.68	4.33
			L	−38	−62	20	5.13	4.05
			L	−28	−74	44	3.95	3.36
	221	Caudate	R	18	−10	22	4.59	3.75
			R	20	−26	18	4.19	3.51
			R	16	−30	10	4.05	3.42
	105	Postcentral Gyrus/S1	L	−36	−28	40	4.72	3.82
			L	−32	−26	48	4.17	3.49
	96	Middle Frontal Gyrus	R	32	14	60	4.37	3.62
			R	30	6	62	3.98	3.37
		Superior Frontal Gyrus	R	24	16	56	4.07	3.43
	84	Middle Temporal Gyrus	L	−42	−50	0	4.40	3.64
			L	−42	−40	−2	3.98	3.38
	69	Supramarginal Gyrus	R	50	−50	30	4.63	3.77
	68	Supramarginal Gyrus	L	−56	−42	24	4.91	3.93
	63	Cuneus	L	−14	−80	20	4.22	3.53

*k*, cluster size in number of voxels, Hem., Hemisphere, L, left, R, right, (-) denotes negative direction, ED = Euclidean distance. Coordinates are in Montreal Neurological Institute (MNI) stereotactic space. *p* < .001 uncorrected, minimal cluster size: 61 voxels.

### Effect of social reactivity on task-based functional activation

**Fig 4:**
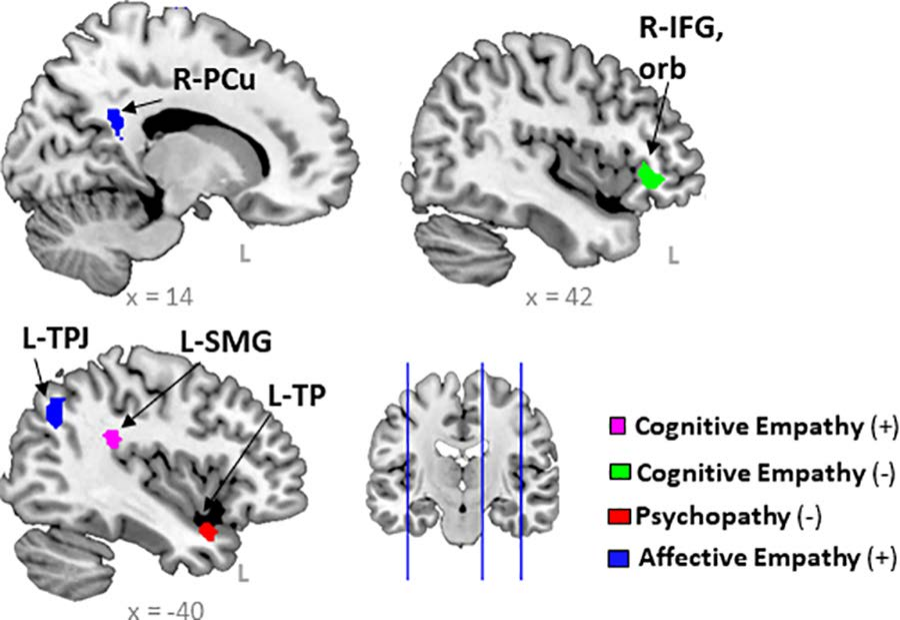
Activation maps: effect of social reactivity on functional activations during social voice modulations. Negative associations between task-based functional activation and cognitive empathy (green) were found in right IFG, orbital part. A cluster in left TP was negatively associated with psychopathy (red). Affective empathy was positively associated with activation in left pTPJ, and PrCu. IFG = inferior frontal gyrus, PCu = precuneus, pTPJ = posterior temporo-parietal junction, L = left, R = right.

Lastly, we explored associations between functional activation during social voice modulation and social reactivity indices. The whole-brain multiple regression model showed a significant negative association between functional activation and cognitive empathy in the orbital part of right inferior frontal gyrus (IFG) and a positive association with functional activation in left supramarginal gyrus (SMG). Affective empathy was positively associated with changes in activation in posterior part of left temporo-parietal junction (TPJ) and right precuneus (PCu), while psychopathy was negatively associated with task-based activation in left temporal pole (TP; uncorrected *p* < .001, *k* = 61, see Table 4, Fig 4). We also conducted an explorative regression analysis of Machiavellianism on functional activation during likeable Go trials based on the behavioral association, which showed a positive association in a cluster in left middle frontal gyrus (MFG) and a negative association in bilateral precuneus (uncorrected *p* < .001, *k* = 60, see S1 Table and S3 Fig).

**Table 4:** Functional activations for the multiple regression whole-brain model of social reactivity traits on Social Go > Rest.

Contrast	*k*	Region	Hem.	X	y	z	*T*	*Z*
(-) Psychopathy	175	Temporal Pole	L	−24	8	−22	5.58	4.15
			L	−40	12	−22	4.63	3.67
			L	−48	12	−18	4.26	3.47
(+) Cognitive Empathy	83	Supramarginal Gyrus	L	−40	−36	26	4.84	3.78
(-) Cognitive Empathy	152	Inferior Frontal Gyrus, orbital part	R	42	32	−4	7.43	4.89
(+) Affective Empathy	139	Posterior Temporo-Parietal Junction	L	−42	−66	36	5.56	3.95
	64	Precuneus	R	14	−46	26	5.17	3.95

*k*, cluster size in number of voxels, Hem., Hemisphere, L, left, R, right, (-) denotes negative direction, (+) denotes positive direction. Coordinates are in Montreal Neurological Institute (MNI) stereotactic space. *P* < .001 uncorrected, minimal cluster size: 61 voxels. Unrelated traits omitted.

## Discussion

In this study, we set out to explore individual differences in the ability to volitionally express social information in the voice. We found that general performance in social vocal control was significantly associated with speakers’ self-reported cognitive empathy, whereas performance in expressing favourable social traits (sounding likeable) was positively associated with Machiavellian traits. On the neural level, individual differences in performance in social vocal control were associated with increased functional activation in areas associated with social processing and vocal motor control. Lastly, we found significant association between social reactivity indices and functional processing during social voice changes that might reflect differential task strategies.

### Association between social reactivity and social vocal control ability

Our behavioural data showed that a speaker’s level of cognitive empathy capacity was significantly associated with general performance in social vocal control, suggesting that the capacity to reflect on others and their thoughts and feelings, contributes to the ability to express trait-related social information vocally. Previous work on perceptual processing in socio-emotional vocal expression has shown that listeners’ social reactivity is an important contributor to successful nonverbal vocal communication, helping to understand the intended social meaning in spoken conversation [11–14]. We show the first evidence that *speakers’* social reactivity might support targeted and fine-tuned vocal behaviour to express social information to others. Specifically, a speaker’s ability to mentalize about others’ thoughts and feelings supports the communication of nuanced social information through the voice. This finding adds to clinical observations, which have shown that spontaneous vocal behaviour in clinical populations with deficits in social reactivity is often characteristically changed or monotonous speech [20,21] and can be used as an implicit source of information about a patient’s social functioning [48]. Nevertheless, it remains unclear which specific social cognitive processes support social voice modulation, and whether this is also true in naturalistic live interaction [49]. In live interactions speakers have to flexibly adjust their vocal behaviour in reference to themselves and the listener. This encompasses both perceptual processing, reactive vocal adjustment and calibration to the interlocutor. Interestingly, previous work measuring linguistic style accommodation in live interactions found no significant effects of empathy on the likelihood of linguistic accommodation [23] – however, in that study nonverbal vocal characteristics were not assessed, and cognitive and affective empathy were not distinguished. We suggest that future studies should examine both spontaneous and volitional vocal behaviour dyads to elucidate the relation between social reactivity (both cognitive and affective empathy), perceptual acuity, and vocal modulation.

Interestingly, the magnitude of the likeable trait percepts evoked by the voice modulations was positively associated with self-reported speaker Machiavellianism (not explained by likeability ratings of the neutral voice, see S2 Text), suggesting that people with a more Machiavellian social style might be particularly efficient in evoking favourable trait impressions on others. This adds to the findings that Machiavellianism is associated with increased vocal convergence to interlocutors, given that it is advantageous for the speaker [23]. One might speculate that speakers who describe their own social interactive style as more Machiavellistic might strategically make use of nonverbal vocal strategies to make favourable impressions on others. More generally, such a charming strategy has previously been associated specifically with people scoring high on Machiavellianism as compared to psychopathy or narcissism [50], and might be a strategy to achieve rapport in others [51]. Interestingly, people scoring high on psychopathic traits on the other hand, have been shown to be very successful in volitionally and convincingly expressing emotions facially to others [24]. Previous work suggests that people scoring high on Machiavellianism also tend to show higher levels of self-monitoring and -control [52,53], and a rational thinking style [54]. These attributes might support the volitional display of emotion in nonverbal behaviours (“posing”; reviewed by [55]), i.e., staying cool while acting out. Of course, more work is needed to disentangle these effects and underlying mechanisms. Exploratory analysis on the neural level showed that a speaker’s level of Machiavelliansim was positively associated with activation in premotor cortex (MFG) during likeable voice modulation, overlapping with an area associated with likeability performance (see S2 and S3 Figs). Simultaneously, cognitive and affective empathy levels were associated with activations in left pTPJ, medial prefrontal cortex and superior temporal sulcus during likeable voice modulation (S2 Table and S4 Fig), suggesting diverging task strategies (see below).

### Neural networks supporting performance in social vocal control

A second aim of this study was to investigate functional activation associated with individual differences in social vocal control. We found that better performance (evoking specific trait percepts in independent listeners) was supported by an increase in activation in left posterior TPJ, bilateral SMG, right premotor cortex, left middle temporal cortex, somatosensory cortex and cuneus, suggesting the involvement of both vocomotor-related and social processing-related brain regions to support specific social trait expressions through the voice. Beyond implicated regions of the VMN (caudate, SMG), sensorimotor cortex and premotor areas in MFG and SFG were positively associated with task performance. This is in line with work showing correlations with performance indices during affective voice modulation [14], association with expertise in pitch control [33] and vocal tract manipulations [34], likely supporting affect-related voice modulation [56]. Performance differences might therefore reflect increased control of vocal operators [57,58] to achieve social voice changes.

SMG in particular supports both auditory and somatosensory feedback integration during speech [59,60], allowing adjustments and monitoring of vocal outputs by matching target and error maps [61]. Acoustic imitation of voices [62] or vocal matching [63] to an internal target engages SMG, particularly when vocal adjustments are voluntary compared to involuntary (e.g., during pitch changes: [64]). Here, we show that activation in SMG is positively associated with more effectual social vocal control performance. In favor of this, engagement of SMG has been shown to vary as a function of experience in singers to achieve pitch changes [33]. We suggest that activation in SMG during social vocal control might effectively support the internal matching to a trait-related voice pattern for motor planning and monitoring. Interestingly, we also found a region in left SMG to be positively associated with individual differences in cognitive empathy during social voice modulations, suggesting a role of SMG beyond isolated audio-motor feedback integration. SMG activation, in fact, might support self-other distinction in complex social tasks [65], such as this one. The region in SMG we report here overlaps with regions associated with specific social cognitive tasks, such as trait judgements or false belief tasks [66]. This implies a dual function of SMG, integrating both social cognitive and vocal motor related processing.

We also identify more posterior areas of angular gyrus/ intraparietal sulcus to be associated with performance in social vocal trait expression. This region corresponds to a more posterior part of temporo-parietal junction (pTPJ). TPJ is a region reliably involved in domain-general cognitive and affective social processing [37,66,67] and left pTPJ is engaged during live conversation [68], volitional vocal impersonations [35], and intention inference from voices [69]. Adding to these findings, our data suggest that the engagement of left pTPJ is predictive of the efficacy with which social traits are expressed in the voice. In our task, most speakers reported imagining a situation in which they had used or would use their voice to express corresponding traits. Left TPJ activation might reflect this process, serving to imagine interactions with specific persons in their lives [70] from a first-person perspective [71,72], through top-down control of endogenous episodic memory retrieval [73]. Other work has shown that left TPJ is essentially involved in monitoring and differentiating self-produced from other-produced speech [74] suggesting a role of TPJ in social feedback monitoring. Indeed, left TPJ has been found to support regulating another’s emotions through vocalizations [75] and in line with this we found that a speaker’s affective empathy was positively associated with activation in left TPJ. Although future studies are needed to determine the exact contribution of these regions to social vocal control, left pTPJ might work together with VMN areas to support encoding social information in the voice [35] by constructing an internal social context to guide vocal behaviour.

In summary, the network of regions reported here might reflect the speakers’ ability to monitor and encode social trait information during ongoing social voice production (pTPJ), through somatosensory and auditory feedback processing (SMG, S1) and vocal motor planning (premotor cortex). We thus offer first evidence that social processing areas, in particular TPJ and SMG, support the ability with which speakers control listeners’ perception of their voice and that processing in these regions is associated with speakers’ trait empathy.

### Differential functional activation associated with social reactivity

We found spatially separable processing regions differentially associated with social reactivity indices, implying different processing strategies might be used to accomplish social voice changes depending on a speaker’s level of these traits. Correspondingly, previous work has suggested that overlapping but separable neural regions might support affective and cognitive social processing [76–79]. The observed results overlap largely with a set of regions that showed increased functional connectivity during live conversation in autistic speakers compared to neurotypical speakers without differences in performance [68]. Our findings add that this might also be the case for nonverbal social vocal behaviour in relation to empathy traits in neurotypical speakers. Given that behavioural performance was significantly related only to cognitive empathy levels, speakers might therefore engage in varying (possibly compensatory) underlying strategies to express social information in the voice, depending on their level of psychopathic, or empathic, processing.

### Considerations and further studies

A limitation of this study is that our sample included a majority of female speakers and raters. Previous work has shown sex differences in some acoustic parameters associated with expressing social traits in the voice [8], as well as specific modulation performance [10]. Although these might be more important in mating contexts, which often rely on exaggerations of sexually dimorphic physical characteristics [80,81], we controlled for effects of sex statistically in all reported analyses. Another limitation is the reliance on self-report measures. However, our instruments showed satisfactory internal consistencies and variation comparable to previous work in non-clinical populations [39,82–84], including specific correlations with informant ratings (peers/spouses) for Machiavellian and psychopathic traits [39].

### Conclusion

Our findings suggest that the success with which speakers can communicate a variety of implicit information about themselves to others is related to their capacity to empathize with others. Further, we find that the efficacy of social vocal control relies on a network of regions supporting fine-tuned motor planning, auditory and somatosensory feedback control and socio-cognitive processing. Lastly, our data indicate differential engagement of these regions depending on a speaker’s dispositional social reactivity.

## Supporting information

S1 Text Acquisition of Imaging Data. All functional brain images were recorded on a 3T Siemens TIM Trio scanner with a 32 channel head coil, using a rapid–sparse event-related 3D echo-planar imaging (EPI) sequence (32 axial slices, slice gap 25%, resolution 3x3x3mm2, flip angle 78°, matrix 64 x 64, TE: 30 msec, TR: 3.5 sec, TA: 2 sec). A 3D T1-weighted MP-RAGE scan was acquired for EPI image alignment and spatial normalization (voxel size 1 mm isotropic; flip angle 11°; TE 3.03 ms; TR 1830 ms; image matrix 256 x 256). Analysis was conducted in SPM12 (http://www.fil.ion.ucl.ac.uk/spm/). Preprocessing steps included spatial realignment, segmentation, co-registration, normalization (functional images were resampled to a voxel size of 2x2x2mm) and smoothing (FWHM = 8 mm). 1st Level general linear models included the vocal control task conditions as regressors and subjective ratings as parametric modulators for each condition.
(DOCX)

S2 Text Correlations between naïve ratings of likeability in neutral voices and Machiavellian Traits. To test for possible baseline effects of Machiavellianism on perception of likeability of speakers’ neutral voices, we did additional post-hoc correlation analysis to compare mean likeability ratings of neutral voices and speakers’ Machiavellianism scores. Partial Pearson correlation analysis revealed no significant association between Machiavellian traits and likeability ratings of neutral voices (*r*_p_ = −.03, *p* = .89) controlling for sex and age.
(DOCX)

S1 Fig Boxplot and descriptive statistics of the social voice modulation index (ED). Higher values indicate worse performance in social vocal control ability, operationalized to worse specificity in evoked trait percepts in listeners. Exemplary RSMs for two speakers are given to illustrate better and worse specificity of evoked trait percepts, reflected in pairwise correlation coefficients in each cell. The minimum of the Euclidean distance (ED) measure would be 0, assuming that a speaker achieves maximal differentiation between evoked trait ratings (the speaker’s RSM and the theoretical RSM would be identical) as the theoretical matrix predicts, whereas 2.45 would be the maximum distance.
(TIF)

S2 Fig Activation maps and descriptive statistics for exploratory functional multiple regression analysis of ∆-Likeability during Likeable Go trials. Activation Maps are shown for Likeability Performance on Likeable Go > Rest together with descriptive statistics (boxplot) of Likeable voice performance (∆-Likeability). Performance in likeable voice modulation was positively associated with functional activation in a cluster in middle frontal gyrus. Only positive correlations survived. The contrast likeable performance (∆-Likeability) on Likeable Go > Rest showed one cluster (*k* = 61) with a peak voxel in left middle frontal gyrus (premotor cortex; MNI coordinates x = −34, y = 10, z = 42, *T* = 5.48, *Z* = 4.23) with an uncorrected *p* < .001 and a minimal cluster threshold of *k* = 60. L = left.
(TIF)

S3 Fig Activation Maps for self-report Machiavellianism and Likeability Performance on Likeable Go > Rest. Machiavellianism was positively associated with functional activation in a cluster in middle frontal gyrus (red), overlapping with the left MFG cluster related to likeability performance (yellow). Machiavellianism was negatively associated with activation in bilateral precuneus (blue). (+/-) denotes positive or negative relationships, respectively. MACH = Machiavellianism, L = left.
(TIF)

S4 Fig Activation maps of social reactivity on functional activations while speaking in a likeable voice. Negative associations between task-based functional activation and cognitive empathy (green) were found in right IFG, orbital part, right STS, left pHC and dmPFC. The cluster in orbital parts of right IFG was also negatively associated with psychopathy (red; overlap shown in yellow). Affective empathy was positively associated with activation in left pTPJ, PrCu, vmPFC. IFG = inferior frontal gyrus, dmPFC = dorsomedial prefrontal cortex, pHC = parahippocampal gyrus, PrCu = precuneus, pTPJ = posterior temporo-parietal junction, STS = superior temporal sulcus, vmPFC = ventromedial prefrontal cortex, L = left, R = right.
(TIF)

S1 Table Table showing exploratory functional activations for multiple regression model of Machiavellianism during Likeable Go trials. The multiple regression model showed a negative association with Machiavellianism in bilateral precuneus (blue) and a positive association with activation in middle frontal gyrus during likeable voice modulations (red; uncorrected *p* < .001, *k* = 25). The cluster in MFG overlapped with a cluster associated with likeable voice performance (∆-Likeability; see S4 Fig).
(DOCX)

S2 Table Table showing effect of social reactivity on functional activations while speaking in a likeable voice. We ran a multiple regression analysis on the contrast Likeable Go > Rest with the questionnaire indices as regressors, while controlling for gender and age. We found no significant clusters associated with Machiavellianism. However, given that this analysis was exploratory, we report associations with all social reactivity indices. Higher affective empathy was associated with increased activation during likeable voice production in the left posterior TPJ, ventral medial prefrontal cortex (mPFC) and precuneus (PrCu). Lower cognitive empathy was associated with increased activation in a cluster in the dorsal mPFC/ anterior cingulate cortex (ACC), right STS, left parahippocampal gyrus (pHC), right orbital inferior frontal gyrus (IFG) and PrCu. Higher psychopathic trait scores were associated with decreased activation in an overlapping region of the orbital IFG, and a region in the temporal pole (uncorrected *p* < .001, *k* = 60).
(DOCX)
