# Supplementary material for: Speaker differences in volitional voice modulation reflected in empathy and functional activation patterns
Source: PLoS One. 2025 Jul 28;20(7):e0325207. doi: 10.1371/journal.pone.0325207 (PMC12303263; doi:10.1371/journal.pone.0325207)
Supplement: S1 Text — All functional brain images were recorded on a 3T Siemens TIM Trio scanner with a 32 channel head coil, using a rapid–sparse event-related 3D echo-planar imaging (EPI) sequence (32 axial slices, slice gap 25%, resolution 3x3x3mm2, flip angle 78°, matrix 64 x 64, TE: 30 msec, TR: 3.5 sec, TA: 2 sec). A 3D T1-weighted MP-RAGE scan was acquired for EPI image alignment and spatial normalization (voxel size 1 mm isotropic; flip angle 11°; TE 3.03 ms; TR 1830 ms; image matrix 256 x 256). Analysis was conducted in SPM12 (http://www.fil.ion.ucl.ac.uk/spm/). Preprocessing steps included spatial realignment, segmentation, co-registration, normalization (functional images were resampled to a voxel size of 2x2x2mm) and smoothing (FWHM = 8 mm). 1st Level general linear models included the vocal control task conditions as regressors and subjective ratings as parametric modulators for each condition. (DOCX) [file pone.0325207.s001.docx]

**S2. Acquisition of Imaging Data.** All functional brain images were recorded on a 3T Siemens TIM Trio scanner with a 32 channel head coil, using a rapid–sparse event-related 3D echo-planar imaging (EPI) sequence (32 axial slices, slice gap 25%, resolution 3x3x3mm2, flip angle 78°, matrix 64 x 64, TE: 30 msec, TR: 3.5 sec, TA: 2 sec). A 3D T1-weighted MP-RAGE scan was acquired for EPI image alignment and spatial normalization (voxel size 1 mm isotropic; flip angle 11°; TE 3.03 ms; TR 1830 ms; image matrix 256 x 256). Analysis was conducted in SPM12 (http://www.fil.ion.ucl.ac.uk/spm/). Preprocessing steps included spatial realignment, segmentation, co-registration, normalization (functional images were resampled to a voxel size of 2x2x2mm) and smoothing (FWHM=8mm). 1st Level general linear models included the vocal control task conditions as regressors and subjective ratings as parametric modulators for each condition.
