# Supplementary material for: Speaker differences in volitional voice modulation reflected in empathy and functional activation patterns
Source: PLoS One. 2025 Jul 28;20(7):e0325207. doi: 10.1371/journal.pone.0325207 (PMC12303263; doi:10.1371/journal.pone.0325207)
Supplement: S2 Text — To test for possible baseline effects of Machiavellianism on perception of likeability of speakers’ neutral voices, we did additional post-hoc correlation analysis to compare mean likeability ratings of neutral voices and speakers’ Machiavellianism scores. Partial Pearson correlation analysis revealed no significant association between Machiavellian traits and likeability ratings of neutral voices (rp = −.03, p = .89) controlling for sex and age. (DOCX) [file pone.0325207.s002.docx]

**S3. Correlations between naïve ratings of likeability in neutral voices and Machiavellian Traits.** To test for possible baseline effects of Machiavellianism on perception of likeability of speakers’ neutral voices, we did additional post-hoc correlation analysis to compare mean likeability ratings of neutral voices and speakers’ Machiavellianism scores. Partial Pearson correlation analysis revealed no significant association between Machiavellian traits and likeability ratings of neutral voices (*r*_p_=-.03, *p*=.89) controlling for sex and age.
