# Supplementary figures and images for: Speaker differences in volitional voice modulation reflected in empathy and functional activation patterns
Source: PLoS One. 2025 Jul 28;20(7):e0325207. doi: 10.1371/journal.pone.0325207 (PMC12303263; doi:10.1371/journal.pone.0325207)

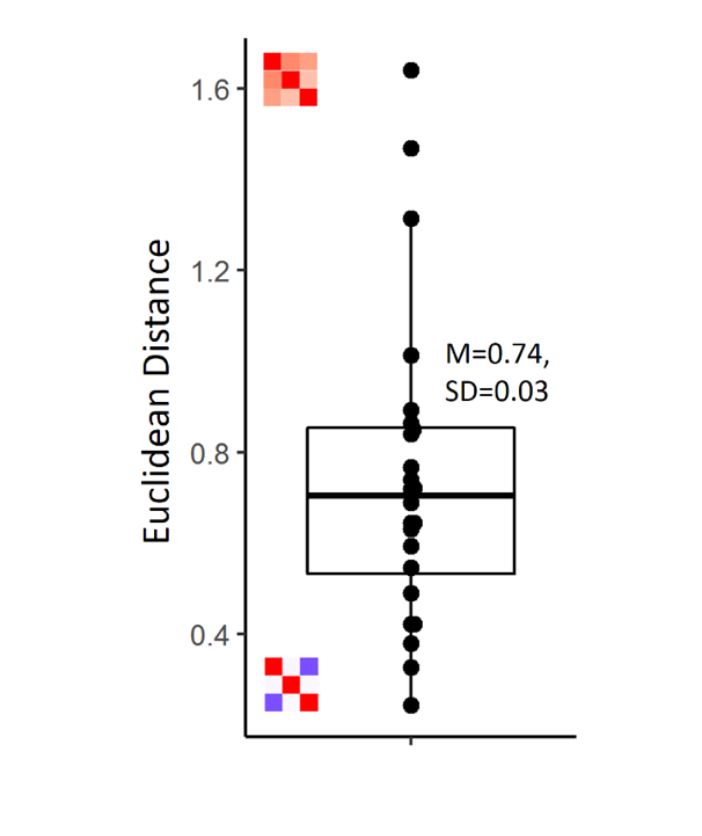

Supplement: S1 Fig — Higher values indicate worse performance in social vocal control ability, operationalized to worse specificity in evoked trait percepts in listeners. Exemplary RSMs for two speakers are given to illustrate better and worse specificity of evoked trait percepts, reflected in pairwise correlation coefficients in each cell. The minimum of the Euclidean distance (ED) measure would be 0, assuming that a speaker achieves maximal differentiation between evoked trait ratings (the speaker’s RSM and the theoretical RSM would be identical) as the theoretical matrix predicts, whereas 2.45 would be the maximum distance. (TIF) [file pone.0325207.s003.tif]

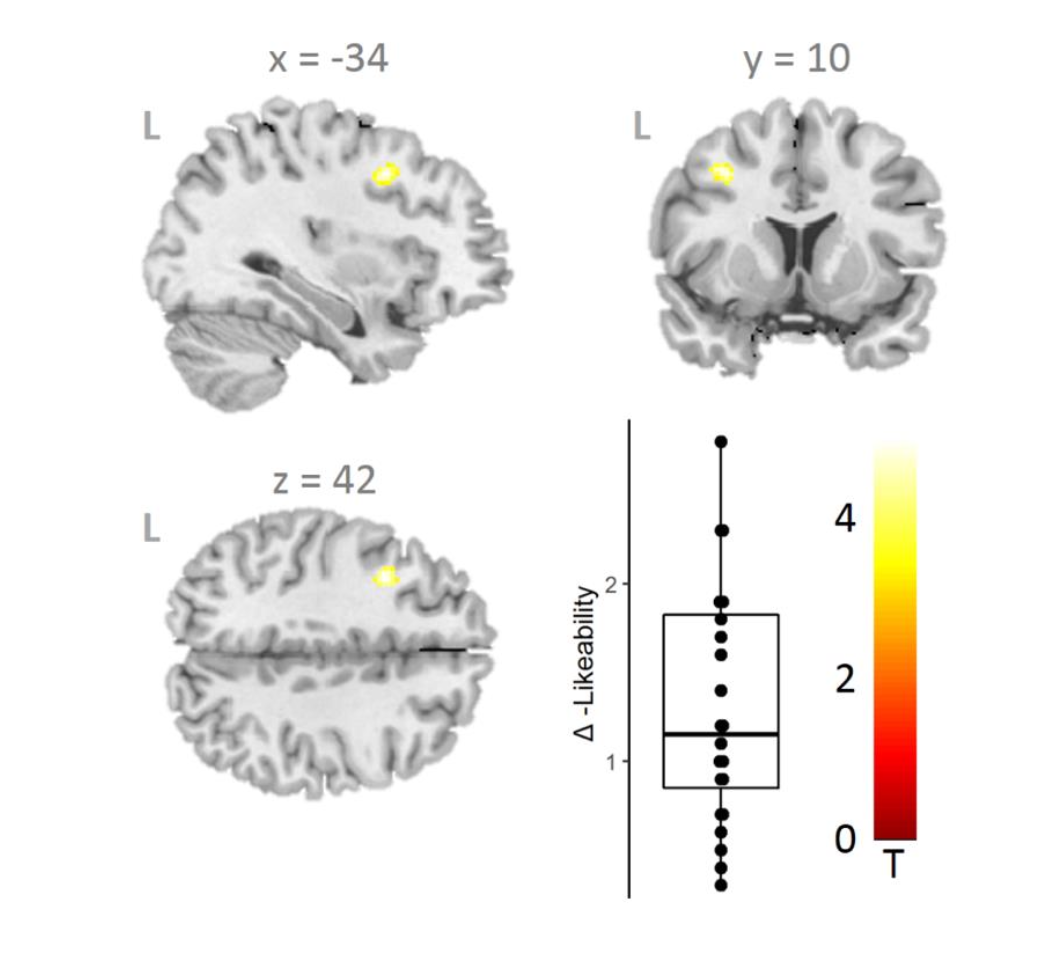

Supplement: S2 Fig — Activation Maps are shown for Likeability Performance on Likeable Go > Rest together with descriptive statistics (boxplot) of Likeable voice performance (∆-Likeability). Performance in likeable voice modulation was positively associated with functional activation in a cluster in middle frontal gyrus. Only positive correlations survived. The contrast likeable performance (∆-Likeability) on Likeable Go > Rest showed one cluster (k = 61) with a peak voxel in left middle frontal gyrus (premotor cortex; MNI coordinates x = −34, y = 10, z = 42, T = 5.48, Z = 4.23) with an uncorrected p < .001 and a minimal cluster threshold of k = 60. L = left. (TIF) [file pone.0325207.s004.tif]

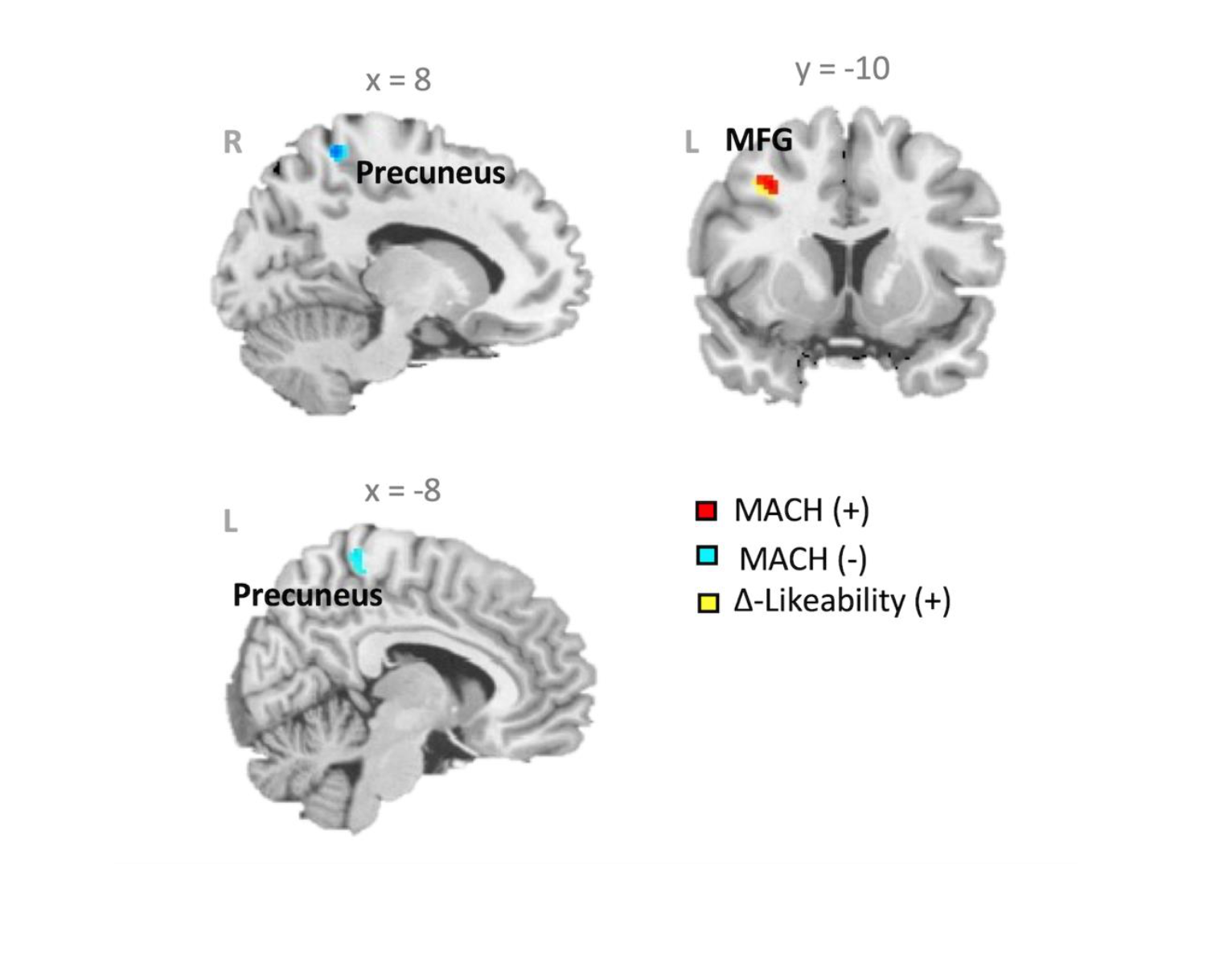

Supplement: S3 Fig — Machiavellianism was positively associated with functional activation in a cluster in middle frontal gyrus (red), overlapping with the left MFG cluster related to likeability performance (yellow). Machiavellianism was negatively associated with activation in bilateral precuneus (blue). (+/-) denotes positive or negative relationships, respectively. MACH = Machiavellianism, L = left. (TIF) [file pone.0325207.s005.tif]

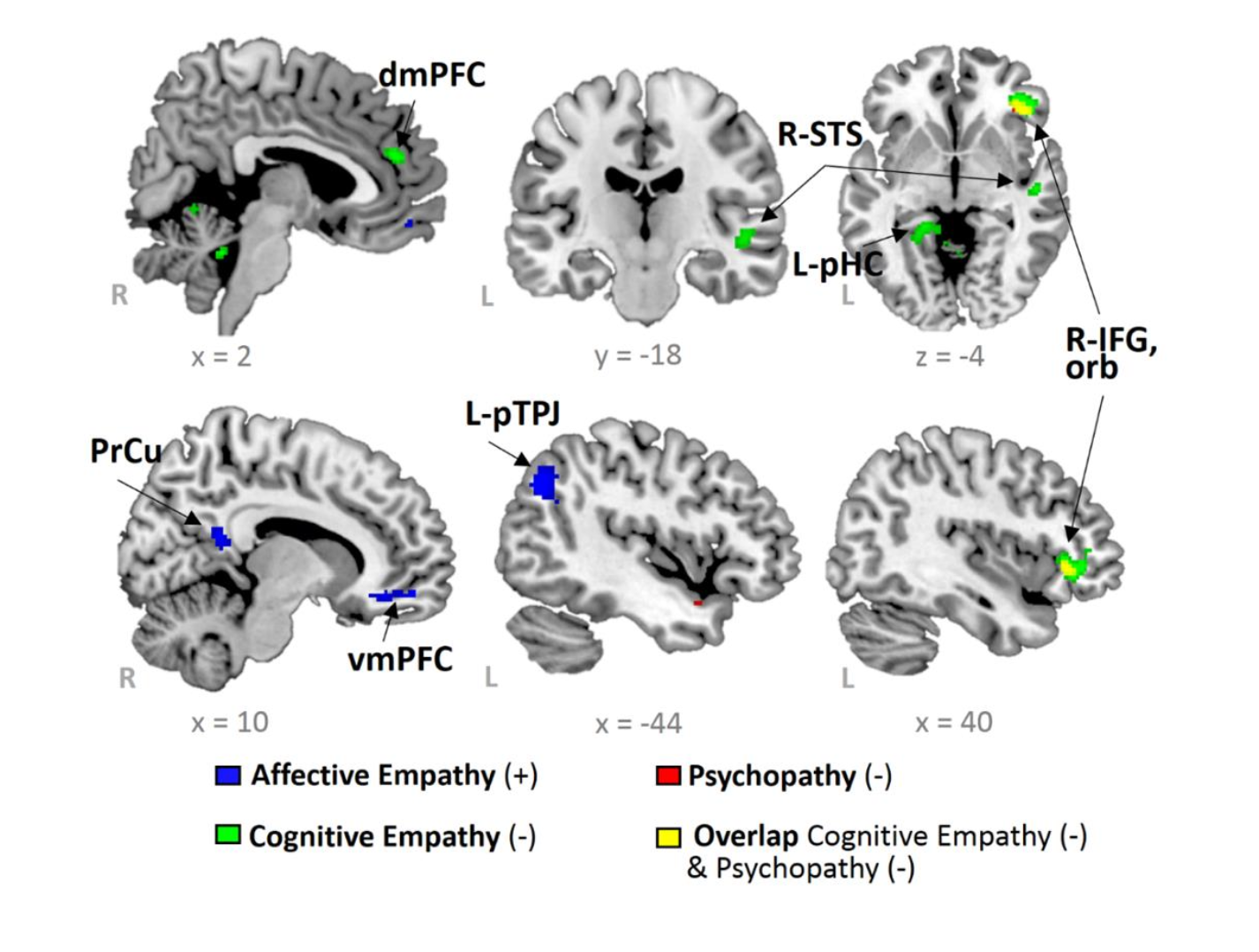

Supplement: S4 Fig — Negative associations between task-based functional activation and cognitive empathy (green) were found in right IFG, orbital part, right STS, left pHC and dmPFC. The cluster in orbital parts of right IFG was also negatively associated with psychopathy (red; overlap shown in yellow). Affective empathy was positively associated with activation in left pTPJ, PrCu, vmPFC. IFG = inferior frontal gyrus, dmPFC = dorsomedial prefrontal cortex, pHC = parahippocampal gyrus, PrCu = precuneus, pTPJ = posterior temporo-parietal junction, STS = superior temporal sulcus, vmPFC = ventromedial prefrontal cortex, L = left, R = right. (TIF) [file pone.0325207.s006.tif]
