# Supplementary material for: Speaker differences in volitional voice modulation reflected in empathy and functional activation patterns
Source: PLoS One. 2025 Jul 28;20(7):e0325207. doi: 10.1371/journal.pone.0325207 (PMC12303263; doi:10.1371/journal.pone.0325207)
Supplement: S1 Table — The multiple regression model showed a negative association with Machiavellianism in bilateral precuneus (blue) and a positive association with activation in middle frontal gyrus during likeable voice modulations (red; uncorrected p < .001, k = 25). The cluster in MFG overlapped with a cluster associated with likeable voice performance (∆-Likeability; see S4 Fig). (DOCX) [file pone.0325207.s007.docx]

| **S5 Table.** **Functional activations associated with Machiavellianism during Likeable Go > Rest.** | | | | | | | | |
| --- | --- | --- | --- | --- | --- | --- | --- | --- |
| **Contrast** | ***k*** | **Region** | **Hem.** | *x* | *y* | *z* | ***T*** | ***Z*** |
| Machiavellianism (-) | 50 | Precuneus | L | -10 | -40 | 66 | 5.90 | 4.44 |
|  | 44 | Precuneus | R | 12 | -48 | 64 | 4.33 | 3.59 |
| Machiavellianism (+) | 43 | Middle frontal gyrus | L | -34 | 10 | 42 | 4.66 | 3.79 |
| *k*, cluster size in number of voxels, Hem., Hemisphere, L, left, R, right. Coordinates are in Montreal Neurological Institute (MNI) stereotactic space.  *p*<.001 uncorrected, minimal cluster size: 25 voxels. | | | | | | | | |
