# Supplementary material for: Speaker differences in volitional voice modulation reflected in empathy and functional activation patterns
Source: PLoS One. 2025 Jul 28;20(7):e0325207. doi: 10.1371/journal.pone.0325207 (PMC12303263; doi:10.1371/journal.pone.0325207)
Supplement: S2 Table — We ran a multiple regression analysis on the contrast Likeable Go > Rest with the questionnaire indices as regressors, while controlling for gender and age. We found no significant clusters associated with Machiavellianism. However, given that this analysis was exploratory, we report associations with all social reactivity indices. Higher affective empathy was associated with increased activation during likeable voice production in the left posterior TPJ, ventral medial prefrontal cortex (mPFC) and precuneus (PrCu). Lower cognitive empathy was associated with increased activation in a cluster in the dorsal mPFC/ anterior cingulate cortex (ACC), right STS, left parahippocampal gyrus (pHC), right orbital inferior frontal gyrus (IFG) and PrCu. Higher psychopathic trait scores were associated with decreased activation in an overlapping region of the orbital IFG, and a region in the temporal pole (uncorrected p < .001, k = 60). (DOCX) [file pone.0325207.s008.docx]

| **Table S6.** **Functional activations for the multiple regression whole-brain model of personality traits on Likeable Go > Rest.** | | | | | | | | |
| --- | --- | --- | --- | --- | --- | --- | --- | --- |
| **Contrast** | ***k*** | **Region** | **Hem.** | **x** | **y** | **z** | ***T*** | ***Z*** |
| (-) Psychopathy | 85 | Temporal Pole | L | -32 | 8 | -22 | 4.49 | 3.59 |
|  |  |  | L | -42 | 10 | -22 | 4.43 | 3.56 |
|  | 71 | Inferior Frontal Gyrus, orbital part | R | 42 | 32 | -4 | 6.20 | 4.42 |
| (+) Cognitive Empathy | 91 | Inferior Parietal lobe | L | -36 | -36 | 28 | 5.52 | 4.12 |
| (-) Cognitive Empathy | 235 | Inferior Frontal Gyrus, orbital part | R | 42 | 32 | -4 | 9.11 | 5.42 |
|  |  |  | R | 42 | 44 | 6 | 4.32 | 3.50 |
|  | 126 | Parahippocampal Gyrus | L | -24 | -46 | -6 | 4.89 | 3.81 |
|  |  | Cerebellum | R | 4 | -58 | -6 | 4.08 | 3.36 |
|  |  |  | L | -10 | -44 | -6 | 4.00 | 3.31 |
|  | 89 | Inferior Frontal Gyrus / Anterior Insula | R | 32 | 14 | -12 | 4.90 | 3.82 |
|  |  |  | R | 32 | 4 | -10 | 3.98 | 3.30 |
|  | 88 | Dorsal Medial Prefrontal Cortex / Anterior Cingulate Cortex | R | 4 | 46 | 20 | 5.43 | 4.08 |
|  | 74 | Cerebellum | R | 4 | -44 | -28 | 5.12 | 3.93 |
|  |  |  | L | -4 | -48 | -32 | 4.29 | 3.48 |
|  | 68 | Precuneus | L | -12 | -56 | 22 | 6.32 | 4.47 |
|  | 65 | Superior Temporal Sulcus | R | 50 | -16 | -4 | 4.89 | 3.81 |
|  |  |  | R | 44 | -24 | 2 | 3.74 | 3.15 |
| (+) Affective Empathy | 236 | Posterior Temporo-Parietal Junction | L | -42 | -66 | 36 | 7.24 | 4.83 |
|  | 93 | Precuneus | R | 12 | -46 | 18 | 5.38 | 4.05 |
|  |  |  | R | 16 | -48 | 28 | 4.51 | 3.60 |
|  | 74 | Ventral Medial Prefrontal Cortex | R | 8 | 50 | -14 | 5.73 | 4.22 |
|  |  |  | R | 12 | 38 | -12 | 4.53 | 3.62 |
| *Note*. *k*, cluster size in number of voxels, Hem., Hemisphere, L, left, R, right, (-) denotes negative direction, (+) denotes positive direction. Coordinates are in Montreal Neurological Institute (MNI) stereotactic space.  *p*<.001 uncorrected, minimal cluster size: 60 voxels. Unrelated traits omitted to increase readability. | | | | | | | | |
